# miR-27b-3p suppresses cell proliferation through targeting receptor tyrosine kinase like orphan receptor 1 in gastric cancer

**DOI:** 10.1186/s13046-015-0253-3

**Published:** 2015-11-14

**Authors:** Jinqiu Tao, Xiaofei Zhi, Xiaoyu Zhang, Min Fu, Hao Huang, Yu Fan, Wenxian Guan, Chen Zou

**Affiliations:** Department of General Surgery, Affiliated Drum Tower Hospital of Nanjing University Medical School, Nanjing, China; Department of General Surgery, Affiliated Hospital of Nantong University, Nantong, Jiangsu China; Division of Gastrointestinal Surgery, Department of General Surgery, Huai’an People’s Hospital, Xuzhou Medical College, Huai’an, Jiangsu China; Department of General Surgery, Affiliated People’s Hospital of Jiangsu University, Zhenjiang, Jiangsu China

**Keywords:** miR-27b-3p, ROR1, Gastric cancer, Cell proliferation, c-Src/STAT3

## Abstract

**Background:**

The receptor tyrosine kinase-like orphan receptors (ROR) family contains the atypical member ROR1, which plays an oncogenic role in several malignant tumors. However, the clinical potentials and underlying mechanisms of ROR1 in gastric cancer progression remain largely unknown. In this study, we validated the microRNA-mediated gene repression mechanism involved in the role of ROR1.

**Methods:**

Bioinformatic prediction, luciferase reporter assay, quantitative real-time PCR (qRT-PCR) and western blotting were used to reveal the regulatory relationship between miR-27b-3p and ROR1. The expression patterns of miR-27b-3p and ROR1 in human gastric cancer (GC) specimens and cell lines were determined by microRNA RT-PCR and western blotting. Cell proliferation, colony formation assay in soft agar *in vitro* and tumorigenicity *in vivo* were performed to observe the effects of downregulation and upregulation miR-27b-3p expression on GC cell phenotypes.

**Results:**

miR-27b-3p suppressed ROR1 expression by binding to the 3’UTR of ROR1 mRNA in GC cells. miR-27b-3p was significantly downregulated and reversely correlated with ROR1 protein levels in clinical samples. Analysis of the clinicopathological significance showed that miR-27b-3p and ROR1 were closely correlated with GC characteristics. Ectopic miR-27b-3p expression suppressed cell proliferation, colony formation in soft agar, xenograft tumors of GC cells. By contrast, miR-27b-3p knockdown enhanced these malignant behaviors. Our studies further revealed that the c-Src/STAT3 signaling pathway was involved in miR-27b-3p-ROR1-mediated cell proliferation regulation.

**Conclusions:**

These results show that miR-27b-3p suppresses ROR1 expression through the binding site in the 3’UTR inhibiting the cell proliferation. These findings indicate that miR-27b-3p exerts tumor-suppressive effects in GC through the suppression of oncogene ROR1 expression and suggest a therapeutic application of miR-27b-3p in GC.

## Background

Gastric cancer (GC) is one of the most common solid malignancies and a leading cause of cancer-related death worldwide [1–6]. In China, GC is the second most diagnosed cause of cancer-related death [7]. Despite great advancements in diagnosis and treatment for this disease, especially surgery, chemotherapy and radiotherapy, the survival remains pessimistic [8]. Accordingly, new clinically applicable molecular targets remain to be discovered for the diagnosis and treatment of GC. The carcinogenesis, development, invasion and metastasis of GC relate to a multi-step and multi-factor complex process, and dysregulation of gene functions participate in this process [4, 9]. Therefore, the identification of novel and specific biomarkers with clinicopathologic and prognostic significance in GC is important.

The receptor tyrosine kinase like orphan receptor 1 (ROR1) is a transmembrane protein that belongs to the receptor tyrosine kinase family. ROR1 consists of an extracellular frizzled-like, cysteine-rich domain, an extracellular, membrane proximal kringle domain and an intracellular tyrosine kinase like domain [10–12]. ROR1 protein is evolutionarily conserved among various species and it is primarily expressed during embryogenesis. The deficiency of ROR1 mice do not display any morphological abnormalities of the skeleton or heart or face, but they die within 24 h after birth probably because of respiratory failure [13, 14]. An increasing number of studies indicated that ROR1 is highly associated with human cancers; however, the biological function of ROR1 remains to be assessed [12, 15–18]. ROR1 may serve as a potential target for cancer therapy [11, 19], and a tumor promoter in a variety solid malignancies, such as breast, lung, ovarian neoplasms. [16, 17] The previous study showed that ROR1 exhibited differential expression between human gastric carcinomas and corresponding normal tissues [20]. These findings indicated that the ROR1 gene might function as an oncogene, but the mechanism by which ROR1 was regulated in GC had yet to be clearly defined. c-Src is one of the most well-characterized protooncogenes and non-receptor protein tyrosine kinases, of which activation is a critical event in the tumorigenesis of a large number of human malignancies, including colon, gastric, breast, lung and prostate cancer [21–23]. Once activated, c-Src is involved in the regulation of normal and oncogenic processes including proliferation, differentiation, survival motility and angiogenesis [21]. c-Src has been shown to interact with several proteins including receptor tyrosine kinases, such as ROR1; and it has previously been demonstrated that ROR1 could enhance the phosphorylation of c-Src; other interaction partners with c-Src, including signal transducers and activators of transcription (STATs), heterotrimeric G proteins, the mitogen-activated protein kinase ERK2, cyclin D, cyclin E and FAK, are important in the cell proliferation and cell cycle regulation [18, 21, 24]. Previous studies suggest that ROR1 tightly involved in cell growth and apoptotic signaling pathways.

MicroRNAs (miRNAs) are highly conserved endogenous small 20–25 nucleotide non-coding RNAs [25, 26]. More than 50 % of the known miRNAs have been shown to participate in human tumorigenesis and/ or metastasis by directly targeting oncogenes or tumor suppressor genes [27, 28]. It is becoming increasingly evident that miRNAs play important roles in the regulation of cell proliferation, apoptosis, migration and invasion thereby affecting normal cell growth and development and leading to a variety of disorders including malignancies [29–31]. Emerging evidences have shown that miRNAs can function as tumor suppressors or stimulators in the tumorigenesis and progression of various human cancers, including gastric cancer [32]. For example, miR-451, miR-101, let-7 and miR-17-92 have been reported to regulate the proliferation, invasion and metastasis of tumor cells by targeting some genes [33–35]. Given that miR-27b has been reported to be downregulated and implicated in suppressing invasion, metastasis in gastric cancer [36]. To the best of our knowledge, limited information is obtainable concerning the clinical potentials and underlying mechanisms of miR-27b-3p in GC thus far. In the current work, we validated that the 3’UTR of ROR1 contains a highly conserved miR-27b-3p binding motif and its direct interaction with miR-27b-3p downregulated endogenous ROR1 protein level. Therefore, this study aimed to investigate the antitumor effect of miR-27b-3p and its correlation with ROR1 in GC cells. We demonstrated the expression of ROR1 and miR-27b-3p in GC cells, validated the relationship between ROR1 and miR-27b-3p, and explored the role of the miR-27b-3p-ROR1 axis in GC cancers. The data showed that miR-27b-3p suppresses cell proliferation by inducing G0/G1 phase arrest mainly through targeting ROR1. Our study is the first report showing the role of miRNA-27b-3p in the regulation of ROR1 expression in GC cells. The results identifying a new tumor-suppressive miR-27b-3p-mediated pathway in GC and provide new insights into the pathogenesis of gastric oncogenesis and will aid the development of novel therapeutic strategies.

## Methods

### Patients and cell lines

The paired tumorous and adjacent non-tumorous human gastric tissues, were obtained from 56 patients with gastric cancer who underwent radical resection in Affiliated People’s Hospital of Jiangsu University. All patients were diagnosed pathologically according to the criteria of the American Joint Committee on Cancer by two professional pathologists independently. All patients gave their informed consent and the study was approved by the institutional ethics committee of Jiangsu University. Written informed consents were obtained before specimen collection. The human GC cell lines MGC803, AGS, MKN45, MKN28, SGC7901, HGC27, NCI-N87 and BGC823, and normal human gastric epithelial cells (GES-1) used in this study were obtained from the Chinese National Human Genome Center at Shanghai.

### Immunohistochemistry

MaxVisionTM techniques (Maixin Bio, China) were used for immunohistochemistry (IHC) analysis, according to the manufacturer’s instructions. After blocking the endogenous peroxides and proteins, 4 μm sections were incubated overnight at 4 °C with diluted primary antibodies specific for ROR1 (Abcam). Next, the slides were incubated with an HRP-Polymer-conjugated secondary antibody at 37 °C for 1 hour. The slides were then stained with a 3,3-diaminobenzidine solution for 3 min and counterstained with hematoxylin. Tumor slides were examined in a blinded manner. Three fields were selected for examination, and the percentage of positive tumor cells and cell-staining intensity were determined.

### Quantitative real-time PCR

Total RNA was extracted with the TRIzol reagent (Invitrogen) according to the manufacturer’s instructions, and was reverse transcribed into cDNA using Primescript RT Reagent (Takara). Real-time PCR was performed using a 7500 Real-time PCR System (Applied Biosystems) with SYBR Premix Ex Taq Kit (Takara). The following primers were used: ROR1, forward: 5’-CAGTCAGTGCTGAATTAGTGCC-3’, reverse: 5’-TCATCGAGGGTCAGGTAAGAAT-3’; β-actin, forward: 5’-AGAGCCTCGCCTTTGCCGATCC-3’, reverse: 5’-CTGGGCCTCGTCGCCCACATA-3’. All procedures were performed in triplicate.

### Luciferase reporter assay

The 3’UTR of ROR1 containing the wild or mutated miR-27b-3p binding sequences were synthesized by Genescript (Nanjing, China). The sequences were cloned into pGL3 luciferase control reporter vector (Promega, USA) to generate the ROR1 3’UTR reporter. Total 5 × 10^5^ cells were seeded in 24-well plates. Cells were cotransfected with 0.12 μg of either pGL3-WT-ROR1 or pGL3-MUT-ROR1 3’UTR reporter plasmid together with 40 nM of miRNA mimics or negative control oligoribonucleotides using Lipofectamine 2000 (Invitrogen) following the manufacturer’s protocol. Firefly and Renilla luciferase activities were measured by Dual-Luciferase reporter assay (Promega). All procedures were performed in triplicate.

### miRNA RT-PCR

Total RNA was extracted as above. Target-specific reverse transcription and Taqman microRNA assay were performed using the Hairpin-itTM miRNAs qRT-PCR Quantitation Kit (Genepharma) according to the manufacturer’s instructions. The reactions were performed on the 7500 Real-Time PCR System. The snRNA U6 was selected as an endogenous reference to calculate the relative expression levels of miR-27b-3p in every sample using the 2^-ΔΔCt^ method. All experiments were done independently in triplicate.

### Vector construct, lentivirus production and cell transfection

miR-27b-3p mimics and miR-27b-3p inhibitor were packaged in lentiviral vector (Genepharma) to overexpress or knockdown miR-27b-3p in GC cells. Lentiviral vector (miR-NC) served as a negative control. The constructed vectors were verified by DNA sequencing. When AGS and BGC823 cells grew to 50 % confluence, cells were infected with lentiviral vectors miR-NC, miR-27b-3p mimics or miR-27b-3p inhibitor, respectively, at an appropriate multiplicity of infection. The above four groups of cells AGS-NC, AGS-miR-27b-3p inhibitor, BGC823-NC and BGC823-miR-27b-3p mimics were selected with 3.5 lg/ml puromycin (Sigma) for 6 days to build stable cell lines. Thereafter, the cells were analyzed using Hairpinit TM miRNAs qRT-PCR Quantitation Kit for miR-27b-3p expression. Stably infected cells were selected for further experiments. The full-length ORF of ROR1 (GeneBank accession no.: NM_005012.3) was amplified from a human GC cell lines cDNA library. The primers were as follows: forward, ROR1-XhoI-F:5’-AGGCTCGAGATGCACCGGCCGCGCCGC-3’, ROR1-HindIII-R:5’-AGGAAGCTTCAGTTCTGCAGAAATCATAGATTC-3’. The PCR product was inserted into the expression vector pcDNA3.1B (Invitrogen). The oligonucleotides encoding short hairpin RNAs (shRNA) were synthesized and inserted into pSUPER vector, and designated pSUPER-shNC-vector (vector), pSUPER-sh-ROR1 (sh-ROR1). Their sequences were: vector, sense, 5’-GATCCCCTTCTCCGAACGTGTCACGT TTCAAGAGAACGTGACACGTTCGGAGAATTTTTGGAAA-3’; antisense, 5’-AGCTTTTCCAAAAATTCTCGAA CGTGTACGTTCTCTTGAAACGTGACACGTTCGGAGAAGGG-3’; sh-ROR1, sense, 5’-GATCCCCCCAGTCACTTATCTGATAATTCAAGAGATTATCAGATAAGTGACTGGTTTTTGGAAA-3’; antisense, 5’-AGCTTTTCCAAAAA CCAGTCACTTATCTGATAATCTCTTGAATTATCAGATAAGTGACTGGGGG-3’.

### Proliferation assay

Cells (2000/well) were seeded into 96-well plates and stained at the indicated time point using the Cell Counting Kit-8 (Dojindo Laboratories), according to the instructions of the manufacturer’s instructions. The optical density measured at 450 nm was used as an indicator of cell viability.

### Colony formation assay

To examine the effect of upregulated or downregulated cortactin expression on proliferation of colon cancer cell lines, cells transfected with different plasmids were used for the colony formation assay. Each type of cell was seeded into 24-well plates (50,000 cells/well) and cultured for 3 weeks in medium containing 1,000 μg/ml G418. These cultures were stained with 0.4 % crystal violet. Clones > 2 mm were counted and the number of clones per well was averaged from three wells for each experiment. Each experiment was repeated at least 3 times.

### Soft agar colony formation assay

The soft agar assay was performed, to evaluate anchorage independent growth. GC cells were resuspended in 0.5 ml 1 % low-melting-point agarose with complete culture medium, and layered on top of 0.5 ml 2 % lowmelting agarose in 24-well plates (2,000 to 5,000 cells/well). The plates were incubated at 37 °C in a humidified atmosphere of 5 % CO2 for 2 weeks. Colonies containing at least 50 cells were counted [37]. All experiments were repeated three times.

### Cell cycle assay

Flow cytometry was used to analyze the cell cycle. First, cells were cultured in serum-free medium for 24 h to induce cell cycle synchronization. Cells were harvested at a different time point. For DNA content analysis, cells were fixed in 70 % ethanol, rehydrated in PBS, treated with RNase A (10 mg/ml) for 30 min then stained with propidium iodide (10 μg/ml) for 5 min.

### Tumor xenografts in vivo

Approximately 4-6 × 10^6^ GC cells were injected subcutaneously into the flank of male nude mice (4 weeks old). Tumors were measured and weighed 4 weeks after injection. Tumor size was measured with calipers every 3 days, and tumor volume was determined using the following formula: Tumour volume = (width^2^ × length)/2.

#### Immunoblotting

Cell extracts were collected in a lysis buffer (50 mM Tris–HCl [pH 7.4], 150 mM NaCl, 1 % Triton X-100, 0.1 % SDS, 1 mM EDTA and protease inhibitor cocktail). The cellular protein was size-fractionated by SDS–polyacrylamide gel electrophoresis and transferred to PVDF membranes (Bio-Rad Laboratories). After blocking with PBS containing 5 % BSA, the membrane was incubated with the appropriate primary antibody at 4 °C overnight, followed by incubation with HRP-conjugated anti-mouse or antirabbit IgG at room temperature for 2 h. The protein bands were detected using an enhanced chemiluminescence (ECL) detection system following the manufacturer’s instructions. The following primary antibodies were used: anti-ROR1 (abcam), anti-c-Src (santa), anti-p-c-Src (santa), anti-STAT3, anti-p-STAT3, anti-cyclinD, anti-c-Myc and anti-GAPDH (cell signaling technology). All procedures were performed in triplicate.

### Statistics

All experiments were repeated in triplicate in this article. Statistical analysis was performed with SPSS software. Data were analyzed using two-tailed Student’s t-tests. Categorical data were evaluated by the χ2 test. Analysis of variance (ANOVA) was used to compare the control and treated groups. *P*-values <0.05 were considered statistically significant.

## Results

### ROR1 is dysregulated in human gastric cancer tissues and cells

**Fig. 1:**
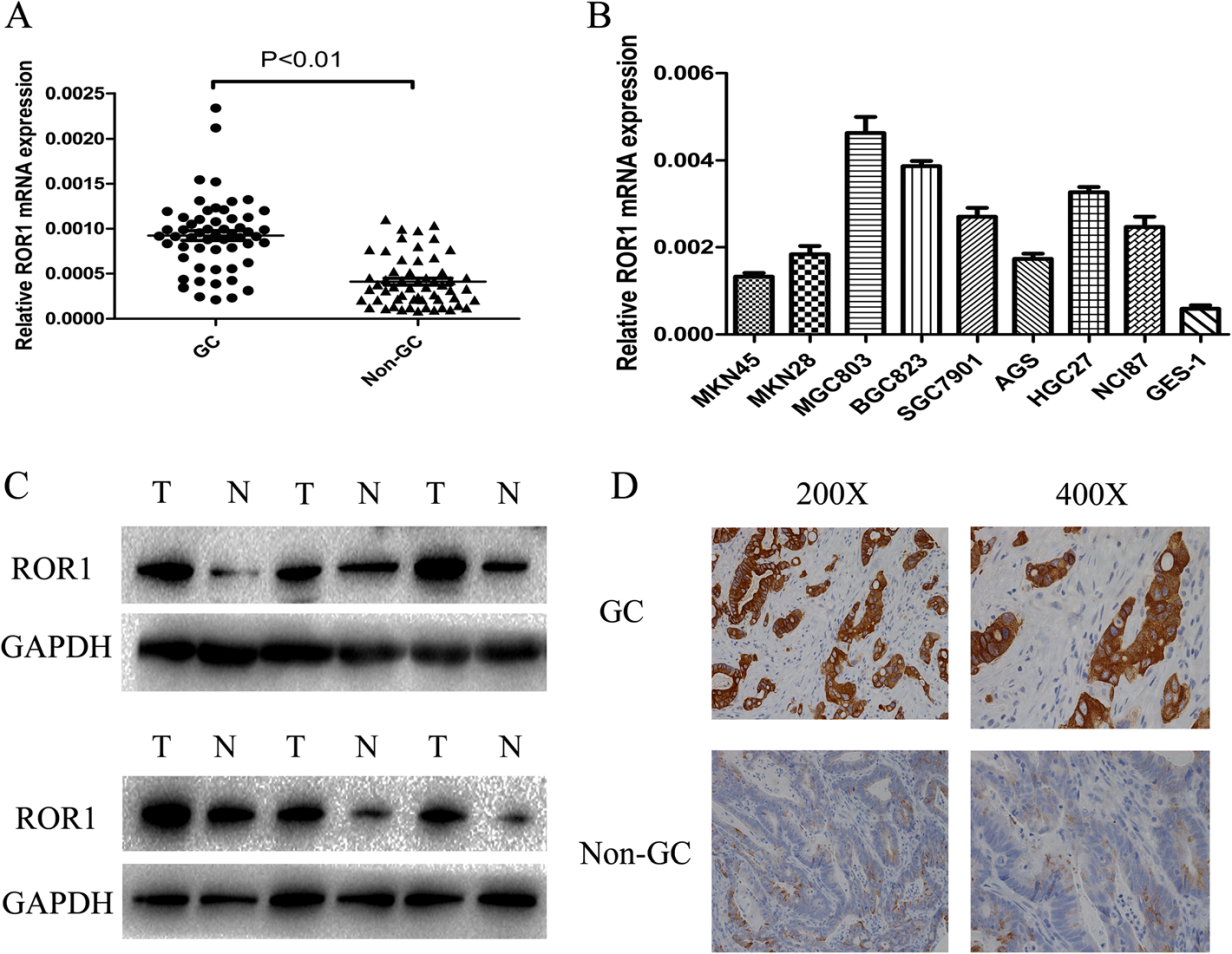
The expression of ROR1 in GC cells. **a** ROR1 mRNA expression in 56 pairs of human GC and their corresponding non-tumour samples. **b** ROR1 mRNA was evaluated in eight GC cell lines and normal human gastric epithelial cells. **c** The protein levels of ROR1 in six paired GC tissues were detected by western blotting. **d** Representative results of the upregulation of ROR1 protein in GC specimens by immunohistochemistry

The expression level of ROR1 was analysed in 56 paired human GC specimens by using real-time PCR. It was shown that the upregulation of ROR1 was significant in the GC tissues compared to the paired adjacent non-tumor samples (Fig. 1a). Furthermore, the expression level of ROR1 in GC cell lines and a normal human gastric mucosal cell line was validated by qRT-PCR (Fig. 1b). We also assessed the protein level of ROR1 in six paired tissues of typical cases by western blotting (Fig. 1c). To further confirm the expression levels of ROR1 in tumor cells, IHC analyze for tumors was employed. The results showed that the expression of ROR1 protein was either high or low in tumor cells, while the ROR1 protein in normal mucosal cells was rarely expressed (Fig. 1d). Furthermore, the correlation between ROR1 or miR-27b-3p expression levels and clinicopathological features were assessed. The patients were split into two groups based on our real time PCR results for ROR1: high expression (+) and low expression (−). It was shown in Table 1 that those patients with tumor size larger than 3 cm had a significantly higher expression of ROR1 than those patients with tumor size smaller than 3 cm. In addition, the expression level of miR-27b-3p was lower in samples with tumor size larger than 3 cm and stages III/IV.

**Table 1:** Expression of miRNA-27b-3p and ROR1 in human gastric cancer according to clinicopathological features of patients

Clinicopathological variables	ROR1 expression		*P* value	miR-27b-3p expression		*P* value
	High	Low		High	Low	
	(*n* = 34)	(*n* =22)		(*n* = 15)	(*n* = 41)	
Age (year)						
<60	14	10		6	19	
≥60	20	12	0.788	9	22	0.767
Gender						
Male	19	14		8	15	
Female	15	8	0.592	7	26	0.359
Tumour size (cm)						
<3	12	16		9	11	
≥3	22	6	0.013*	6	30	0.030*^*^
Differentiation						
Well	10	9		10	22	
Moderately and poorly	24	13	0.401	5	19	0.544
Stage						
I + II	13	7		8	9	
III+ IV	21	15	0.777	7	32	0.046*

*P < 0.05 Statistically significant difference

### ROR1 knockdown inhibits cell proliferation and induces cell cycle arrest

**Fig. 2:**
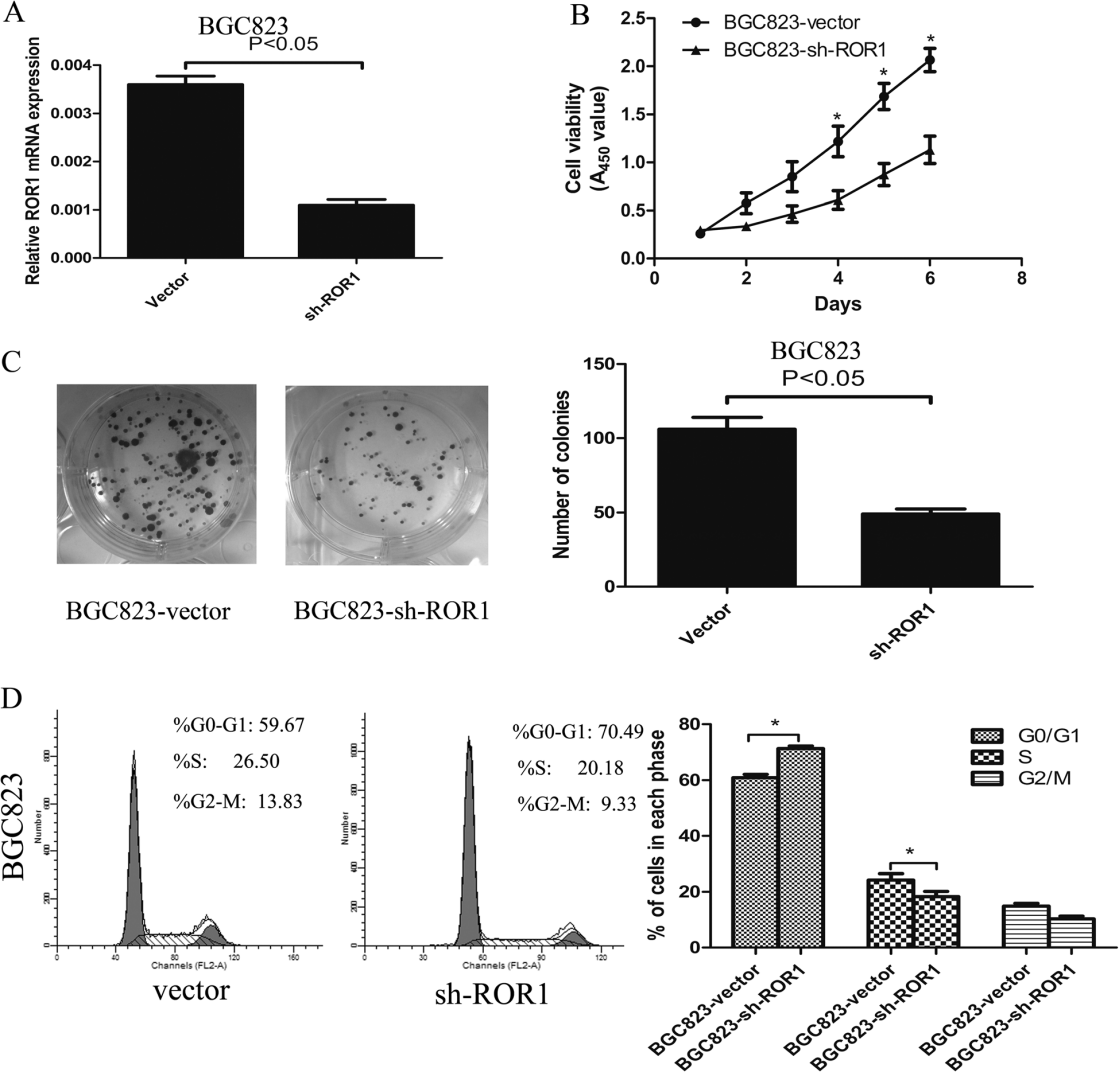
ROR1 regulates cell proliferation, colony formation and cell cycle *in vitro*. **a** and **b** Cells were transfected with vector and sh-ROR1, and the knockdown efficiencies of sh-ROR1 in BGC823 cell were measured by RT-PCR, and the different symbols represent the corresponding mean values of the experiments, performed in triplicate (mean ± SD), *P* < 0.05 was calculated on days 4, 5 and 6; **c** Knockdown of ROR1 inhibits colony formation of BGC823 cells. **d** The effect of ROR1 on cell cycle

pSUPER-sh-NC-vector (vector) and pSUPER-sh-ROR1 (sh-ROR1) were built in order to knockdown endogenous ROR1 in BGC823 cell line. qRT-PCR was used to determinate the expression of ROR1. CCK-8 assay and colony formation were used to evaluate proliferative ability. Flow cytometry was used to measure cell cycle distribution. The expression of ROR1 was downregulated by sh-ROR1 transfection (Fig. 2a). Further, ROR1 knockdown suppressed cell proliferative viability (Fig. 2b and c). In addition, ROR1 knockdown induced G0/G1 phase arrest (Fig. 2d). These data suggest that therapeutic ROR1 knockdown may favor the prognosis of GC patients.

### ROR1 is a direct downstream target of miR-27b-3p

**Fig. 3:**
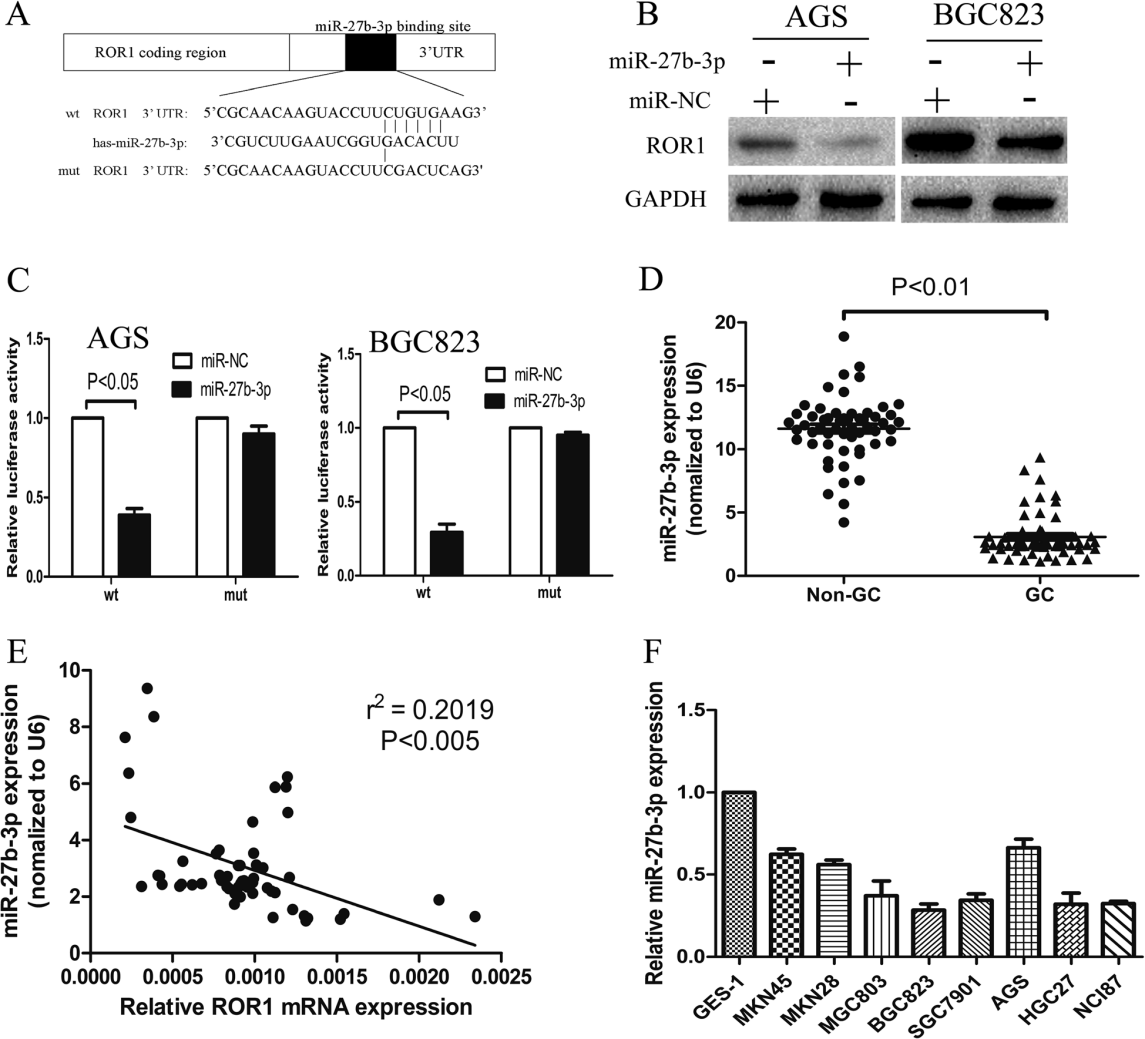
ROR1 is a direct downstream target gene of miR-27b-3p. **a** The ROR1 3’UTR regions containing the wild-type or mutant binding site for miR-27b-3p are shown. **b** Western blotting was used to analyze the expression levels of ROR1 after transfecting with miR-NC or miR-27b-3p mimics. **c** Relative ROR1 luciferase activity was analyzed after the wild-type or mutant 3’UTR reporter plasmids co-transfected with miR-NC or miR-27b-3p. **d** RT- PCR was used to detect the expression levels of miR-27b-3p in GC and non-GC specimens. **e** The scatter plots show the expression levels of miR-27b-3p and ROR1 in GC samples. Linear regression analysis was used to measure the association between miR-27b-3p and ROR1. **f** The expression of miR-27b-3p in a panel of tumorigenic GC cell lines

Previous study suggested that ROR1 is highly upregulated in GC tissues led us to explore the mechanism of ROR1 overexpression [18]. To investigate whether the expression of ROR1 is regulated by miRNAs, TargetScan (http://www.targetscan.org/), PicTar (http://pictar.mdc-berlin.de/) and miRanda (http://www.microrna.org/microrna/home.do) were used in combination to predict miRNAs which might target ROR1. As shown in Fig. 3a, we found that miR-27b-3p can bind the 3’UTR of ROR1. The luciferase reporter assay was employed to validate the hypothesis. Wild-type and mutant ROR1 3’UTR containing putative target sites of miR-27b-3p were cloned into reporter plasmids respectively. The results revealed that the activity of the luciferase reporter gene fused to the ROR1 3’UTR was significantly declined by miR-27b-3p. Luciferase responsiveness to miR-27b-3p was abrogated because of the mutation of the putative miR-27b-3p binding sites in the 3’UTR of ROR1 (Fig. 3c). Western blotting showed that ROR1 protein levels were inhibited in miR-27b-3p mimics-transfected cells (Fig. 3b). Furthermore, we confirmed that miR-27b-3p was significantly downregulated in GC and GC cell lines (Fig. 3d and f), notably, by analyzing of their expression levels in GC tissues, the expression level of miR-27b-3p was found to be negatively associated with ROR1 mRNA expression (Fig. 3e). In summary, ROR1may be as an oncogene accelerator in GC.

### miR-27b-3p suppressed cell proliferation and induce cell cycle arrest mainly by targeting ROR1 in vitro

**Fig. 4:**
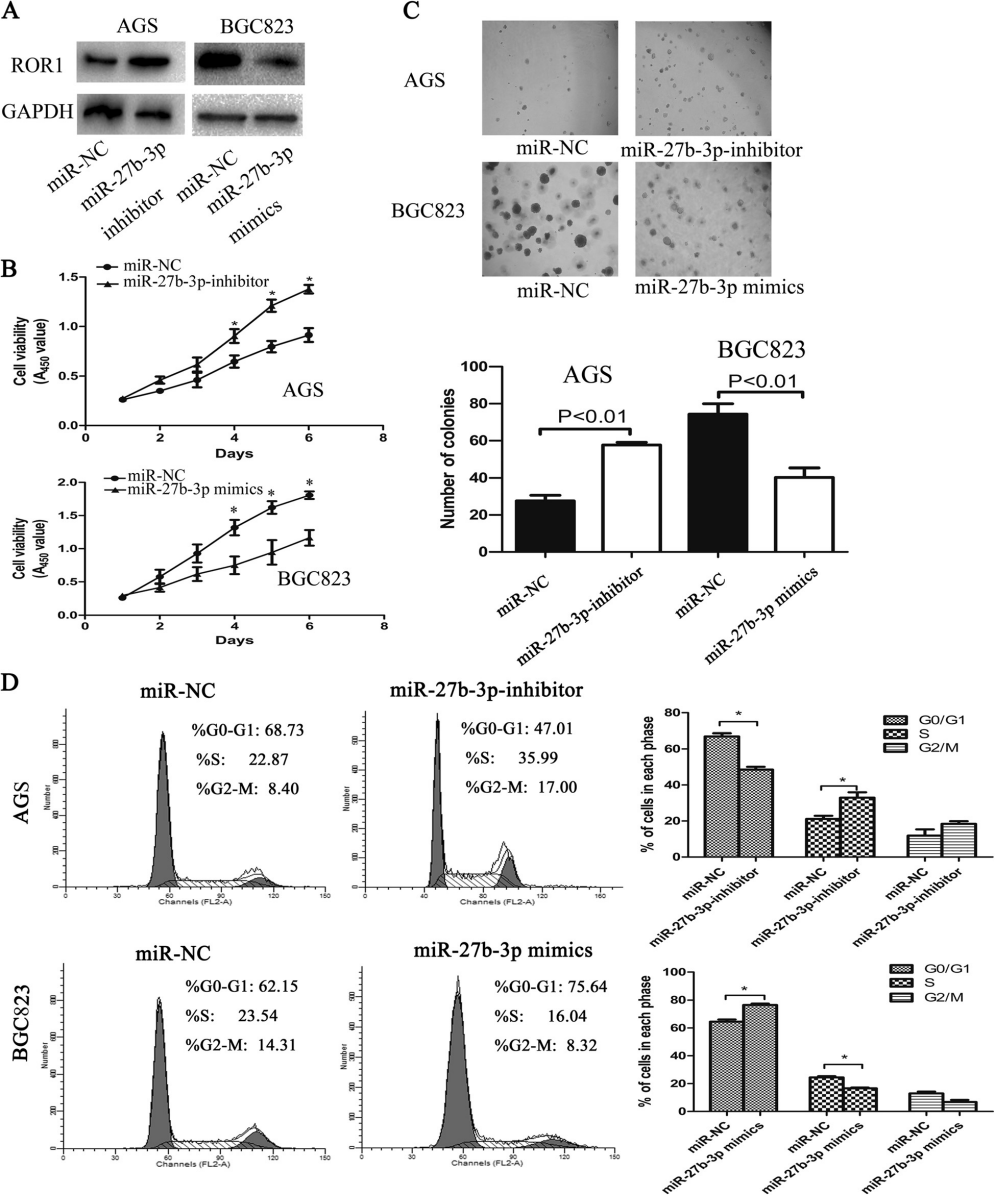
miR-27b-3p inhibits GC progression *in vitro*. **a** Western blotting assay was used to analyze the expression levels of ROR1 and miR-27b-3p in AGS and BGC823 cells transfected with miR-NC, miR-27b-3p inhibitor or miR-27b-3p mimics; **b** Representative profiles of CCK-8 cell growth in AGS cells after transfection with miR-27b-3p inhibitor compared to the miR-NC, transfection with miR-27b-3p mimics inhibited cell proliferation compared with miR-NC used as control. **c** Effects of miR-27b-3p on the colony formation of GC cells. The number of colonies was calculated and analyzed. **d** The effects of miR-27b-3p on cell cycle

**Fig. 5:**
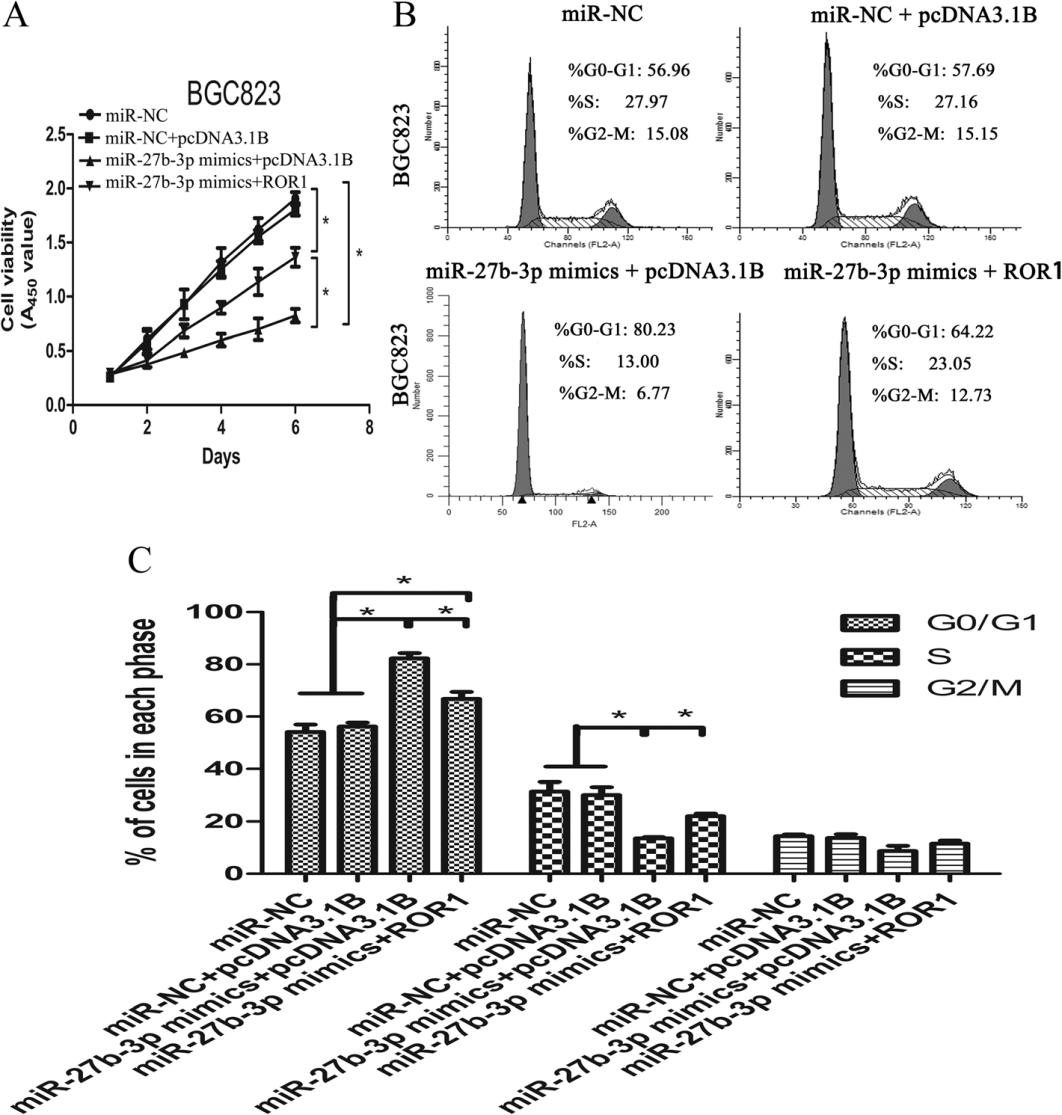
miR-27b-3p inhibits GC progression by mainly downregulating ROR1 *in vitro*. **a** Cells were transfected with miR-NC, miR-NC + pcDNA3.1B, miR-27b-3p mimics + pcDNA3.1B, miR-27b-3p mimics + ROR1, respectively, and cell viability was measured by the CCK-8 assay at 1 d, 2 d, 3d, 4d, 5d and 6d; **b** and **c** Cell cycle distribution was determined by flow cytometry analysis. Mean ± SD, *p < 0.05

To determine the role of the direct interaction between miR-27b-3p and ROR1, western blotting was used. As shown in Fig. 4a, miR-27b-3p could inhibit the protein levels of ROR1 in BGC823 cell line. In contrast, the miR-27b-3p inhibitor upregulated ROR1 protein levels in AGS cell line. Furthermore, cell proliferation was measured by the CCK-8 and colony formation in soft agar assay, and miR-27b-3p significantly suppressed cell proliferation in BGC823 cell line, whereas miR-27b-3p inhibitor enhanced cell viability and colony formation (Fig. 4b and c). In addition, flow cytometry indicated similar cell cycle distribution in cells with overexpression of miR-27b-3p through induction of G0/G1 phase arrest. miR-27b-3p inhibitor was further used in AGS cell, the results showed that miR-27b-3p is downregulated by specific inhibitors, along with cell cycle progression (Fig. 4d). To further illustrate miR-27b-3p that affects cell proliferation by regulating ROR1, we investigated whether ROR1 counteracted the suppression of cell phenotypes caused by miR-27b-3p overexpression in GC cells. The vector ROR1, which contains only the ROR1 coding sequence, was constructed for ROR1 expression without miR-27b-3p targeting. BGC823 cells were cotransfected with miR-27b-3p mimics and either ROR1 or pcDNA3.1B empty vector. The data clearly confirmed that ectopic expression of ROR1 partly reversed the suppression of cell proliferation and cell cycle arrest caused by miR-27b-3p overexpression (Fig. 5a, b and c). These data collectively indicated that miR-27b-3p inhibits cell proliferation and induces cell cycle arrest mainly by targeting ROR1, and that miR-27b-3p may act as a “tumor suppressor” in GC.

### miR-27b-3p influences tumorigenesis and tumor burden

**Fig. 6:**
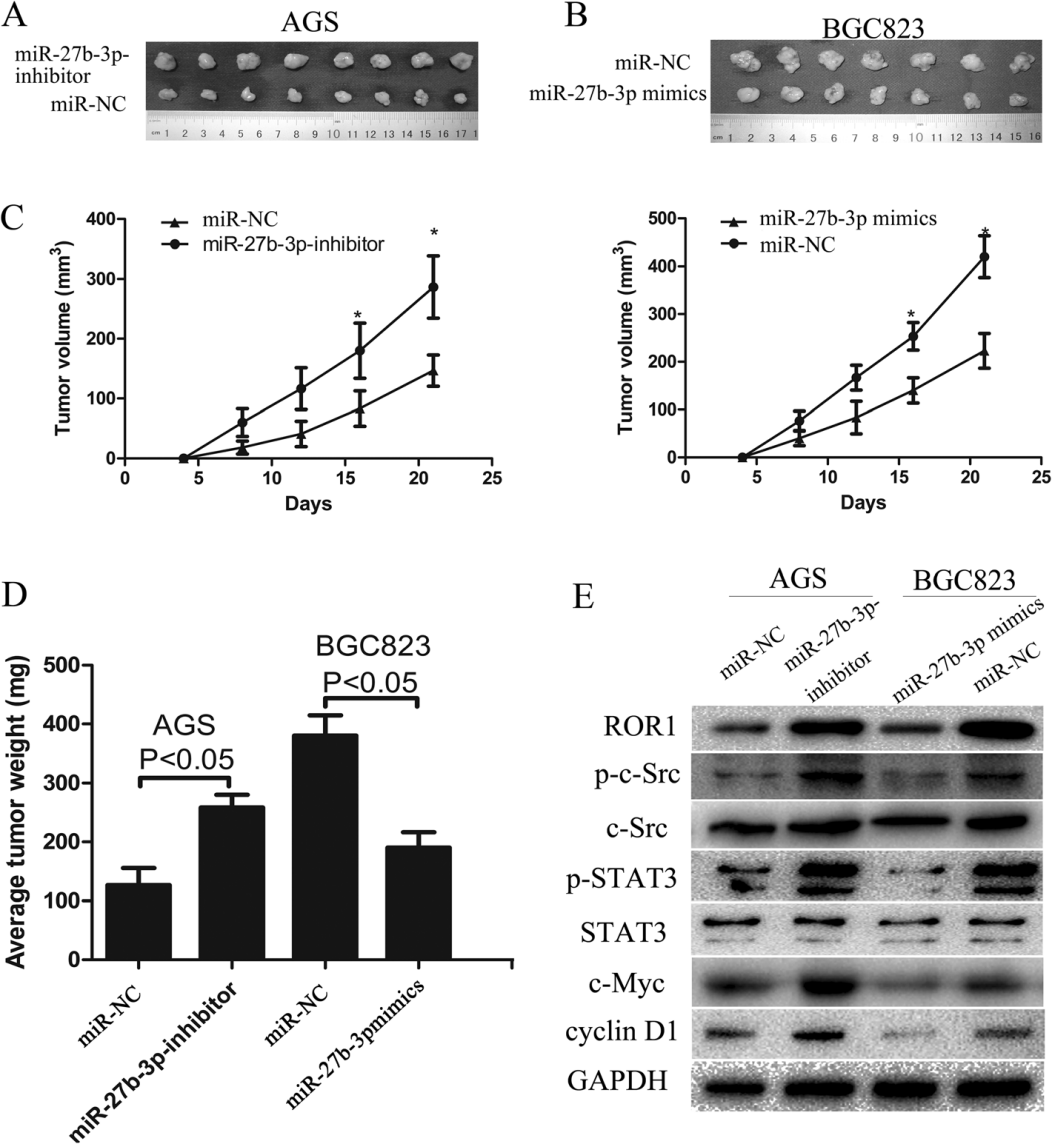
miR-27b-3p inhibits xenograft tumor growth of GC cells. **a** and **b** Photographs of tumors derived from the different groups of nude mice; the graph is representative of tumor growth 21 days after inoculation. **c** and **d** Tumor volume and the weight were calculated, and all date are shown as mean ± SD (* *P* < 0.05). **e** Western blotting analysis of c-Src/STAT3 signaling pathway related proteins showed upregulation of p-c-Src, p-STAT3, c-Myc and cyclin D1 in miR-27b-3p inhibitor of AGS cell and downregulation of p-c-Src, p-STAT3, c-Myc and cyclin D1 in miR-27b-3p mimics of BGC823 cell, total c-Src and total STAT3 protein level was unchanged

The effects miR-27b-3p on the tumourigenic potential of GC cells in vivo were also evaluated. Compared with AGS cells transfected with miR-NC, injection of the same cells transfected with miR-27b-3p-inhibitor resulted in larger tumors sizes in male nude mice about 4 weeks after injection (Fig. 6a, c and d). To determine the effect of overexpression of miR-27b-3p on tumorigenicity *in vivo*, miR-27b-3p mimics was used to transfect BGC823 cells, then these cells were harvested and injected into the flank of male nude mice. As expected, miR-27b-3p mimics was able to significantly suppress tumorigenicity, resulting in obvious reductions in tumor weight and volume compared to miR-NC (Fig. 6b, c and d). All these data indicate that miR-27b-3p mediated ROR1 plays a key role in GC tumorigenesis.

### miR-27b-3p mediated differential expression of ROR1 affects the c-Src/STAT3 signaling pathway related proteins

To further study the mechanism by which miR-27b-3p *mediated* ROR1 enhances the growth of GC, the c-Src, p-c-Src, STAT3, p-STAT3, c-Myc and cyclin D1 protein levels were detected by western blotting. The levels of p-c-Src, p-STAT3, c-Myc and cyclin D1 were shown to be significantly upregulated in AGS cell line transfected with miR-27b-3p-inhibitor compared with the same cells transfected with the miR-NC, however, no difference in the total c-Src and STAT3 protein levels were observed between the two cell groups. As expected, a reduction in the level of p-c-Src, p-STAT3, c-Myc and cyclin D1 protein in BGC823 was caused by the miR-27b-3p mimics compared with the negative control, likewise, the level of total c-Src and total STAT3 expression were found to be almost unchanged (Fig. 6e). These data indicate that miR-27b-3p mediated differential expression of ROR1consequently resulted in activation of c-Src/STAT3 signaling pathway.

## Discussion

Although there is extensive information from the past about gastric cancer at genetic and molecular level, differing clinical courses and the limited value of established prognostic markers have compelled researchers to look for new molecular parameters in predicting the prognosis and treatment of patients with GC. Identifying appropriate molecular targets and understanding the molecular basis of these pathways is an important step.

Recently, aaccumulating evidence suggests that the aberrant miRNAs expression signature is a hallmark of malignancies, it also has been reported that miRNAs play important roles in regulating diverse cellular processes including proliferation, apoptosis, migration and invasion [29–31, 38]. We can infer that, to date, the mechanism by which miRNA exerts its function is still a topic of great interest in cancer biology. Although many studies have reported the role of miR-27b in cancer progression, much remains to be illuminated to supplement the network of its interactions, a series of comprehensive research data have identified miR-27b as a tumor suppressor in a series of malignant tumor [36, 39, 40]. However, limited information is obtainable concerning the clinical potentials and underlying mechanisms of miR-27b-3p in GC thus far. Herein, we demonstrated that miR-27b-3p could regulate cell proliferation, colony formation and tumorigenicity by targeting oncogene ROR1 in GC. In our previous study, we used bioinformatics software to predict the candidate miRNAs targeting ROR1. Bioinformatic prediction, luciferase reporter assay, qRT-PCR and western blotting were used to reveal the regulatory relationship between miR-27b-3p and ROR1. In this study, our data also demonstrated that miR-27b-3p could repress ROR1 protein expression in GC cells. Furthermore, compelling evidences proved that miR-27b-3p was significantly downregulated and reversely correlated with ROR1 protein levels in clinical samples. Herein, our findings conclude that ROR1 could be a new target gene of miR-27b-3p in GC.

ROR1 is a member of the RORs family which consists of ROR1 and ROR2. RORs contain two distinct extracellular cysteine rich domains and one transmembrane domain. RORs are transmembrane proteins which are members of the receptor tyrosine kinase family. The intracellular part of ROR1 possesses a tyrosine kinase domain, two serine/threonine-rich domains and a proline-rich domain [10, 41]. The ROR1 is shown to play a role in tumor-like behavior, such as cell migration and cell invasiveness and are negatively expressed in normal adult tissue, ROR1 has recently been found to be expressed in human cancers, and have the potential to be cancer targets [11, 17]. Ectopic expression of ROR1 is also observed in a wide variety of solid malignancies, tissue microarray analysis showed strong staining of ROR1 in 30 % or greater of primary samples in colon, lung, and pancreatic cancers [16]. However, moderate staining is detected in the majority of ovarian, lymphoma, skin, testicular, uterine, prostate, and adrenal cancers [16]. In this study, ROR1 was identified as an important downstream target of miR-27b-3p. Overexpression of miR-27b-3p significantly reduced the ROR1 level in GC cells, and the inhibitory effects of miR-27b-3p on GC cell proliferation and colony formation were reversed by overexpression of ROR1. Furthermore, ROR1 was increased in GC tissues and negatively correlated with the expression of miR-27b-3p. Taken together, these results indicated that miR-27b-3p might suppress cell proliferation through targeting ROR1.

c-Src is one of the most well-characterized protooncogenes and non-receptor protein tyrosine kinases. In fact, c-Src is known to be overexpressed and/or hyper-activated in a wide variety of human cancers, such as colon, gastric and prostate cancer [22, 23, 42]. c-Src plays a role as transcriptional regulator and enhances carcinogenesis via regulating a series of downstream target genes. However, the biological effects of this gene in GC development have are still unclear.

Recently c-Src has been identified as a substrate gene of ROR1, which is capable of interacting with c-Src and control c-Src-mediated transformation, and could further participate in neoplastic transformation and contribute to tumorigenesis [24, 43]. This result suggests that ROR1 may promotes the proliferation of cancer cells through c-Src activation. The signal transducer and activator of transcription family was important for promoting the proliferation, survival and other biological processes triggered by cytokines and growth factors [44–47]. Although there are seven STAT proteins, STAT3 is the most important downstream molecule for c-Src kinase [48–50]. The increased protein level of phosphorylation c-Src (p-c-Src) in gastric cancer cell caused by ROR1 was identified in this study. Given that ROR1 can enhance the phosphorylation of c-Src, other downstream target genes which include STATs, heterotrimeric G proteins, the mitogen-activated protein kinase ERK2, cyclin D, cyclin E, c-Myc and FAK, are the important key point in the cell proliferation [18, 21, 24]. Therefore, the relationship between ROR1 expression in GC cells and cell cycle was analyzed. We found that ROR1 promotes phase transition of GC cells into S phase from G0/G1 phase. It has been reported in several studies that regulation of G1/S phase transition is often abnormal in cancer cells, with the cyclins or CDK proteins and their upstream regulators changed accordingly [51–54]. Cyclin D1 is the major cyclin regulating cell cycle transition from G0/G1 phase to S phase [55, 56]. In this study, our data indicate that ROR1 is the main downstream target gene of miR-27b-3p, and that miR-27b-3p can bind to its 3’UTR and subsequently regulate its expression post-transcriptionally. Re-expression ROR1 upon miR-27b-3p overexpression could partly reverse but not completely prevent the effect of miR-27b-3p. Thus, miR-27b-3p additionally suppresses cell proliferation via interactions with other target genes, although miR-27b-3p suppresses cell proliferation and induces G0/G1 cell cycle arrest by mainly targeting ROR1.

## Conclusions

**Fig. 7:**
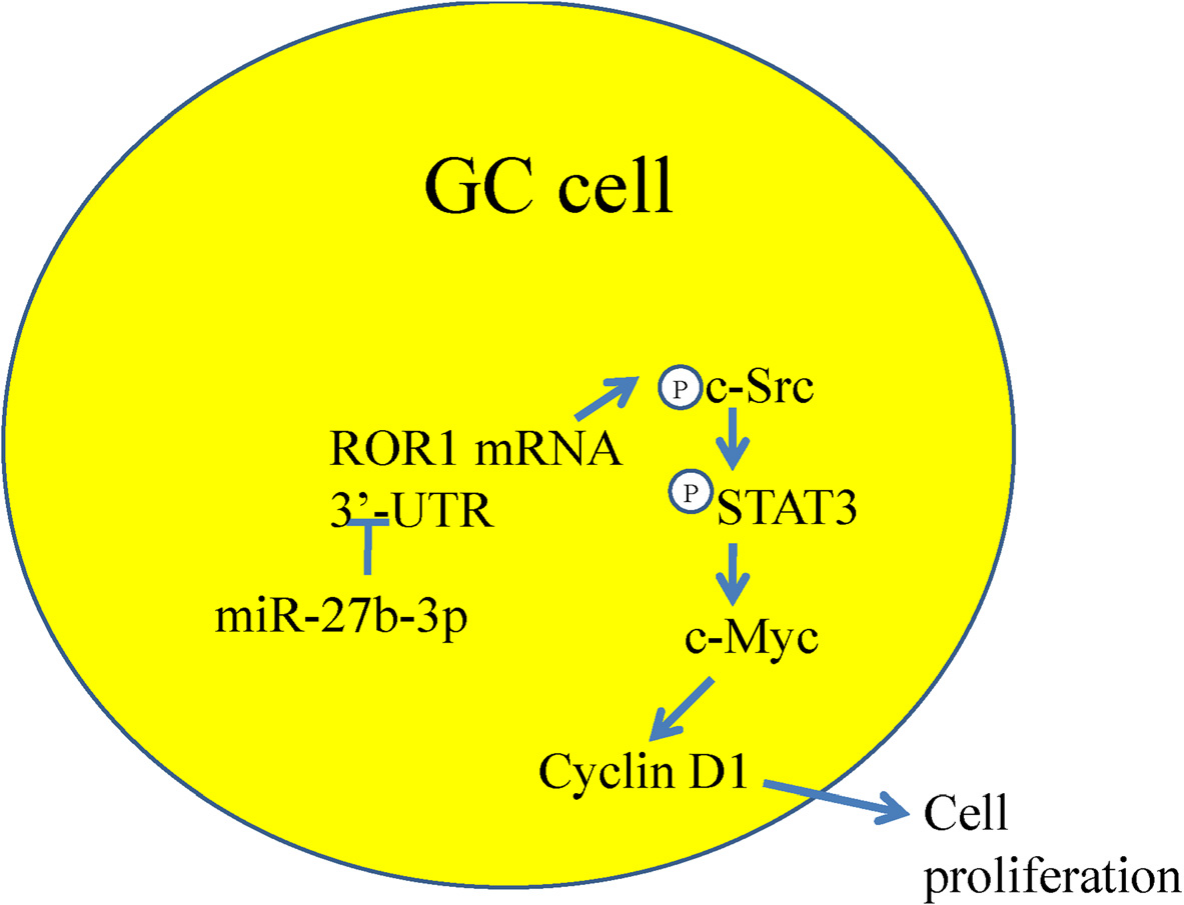
Schematic miR-27b-3p inhibits GC cell proliferation through ROR1 suppression.

In conclusion, the present study provides evidence that the dysregulation of ROR1 results from miR-27b-3p downregulation in GC and may promote cancer progression, and the c-Src/STAT3 signaling pathway was involved in miR-27b-3p-ROR1-mediated cell proliferation regulation (Fig. 7). Therefore, this study demonstrates a novel regulator of ROR1 and enriches our knowledge on the interactions between miR-27b-3p and its targets in GC. Thus, our findings provide new prospects for miR-27b-3p and ROR1 as promising molecular therapies in GC treatment.

## Abbreviations

ROR1: is short for receptor tyrosine kinase like orphan receptor 1
GC: is short for Human gastric cancer
3’UTR: is short for 3’-untranslated region
RT-PCR: is short for reverse transcription polymerase chain reactions
